# Distinct processing of tone offset in two primary auditory cortices

**DOI:** 10.1038/s41598-019-45952-z

**Published:** 2019-07-03

**Authors:** Magdalena Sołyga, Tania Rinaldi Barkat

**Affiliations:** Brain & Sound Lab, Department of Biomedicine, Basel University, 4056 Basel, Switzerland

**Keywords:** Cortex, Sensory processing

## Abstract

In the rodent auditory system, the primary cortex is subdivided into two regions, both receiving direct inputs from the auditory thalamus: the primary auditory cortex (A1) and the anterior auditory field (AAF). Although neurons in the two regions display different response properties, like response latency, firing threshold or tuning bandwidth, it is still not clear whether they process sound in a distinct way. Using *in vivo* electrophysiological recordings in the mouse auditory cortex, we found that AAF neurons have significantly stronger responses to tone offset than A1 neurons. AAF neurons also display faster and more transient responses than A1 neurons. Additionally, offset responses in AAF – unlike in A1, increase with sound duration. Local field potential (LFP) and laminar analyses suggest that the differences in sound responses between these two primary cortices are both of subcortical and intracortical origin. These results emphasize the potentially critical role of AAF for temporal processing. Our study reveals a distinct role of two primary auditory cortices in tone processing and highlights the complexity of sound encoding at the cortical level.

## Introduction

For sound perception, auditory signals have to travel a long way from the cochlea, through the cochlear nuclei, superior olivary complex, inferior colliculus, and auditory thalamus up to the auditory cortex where two regions, A1 and AAF, receive them in parallel. An early study already distinguished A1 and AAF as two separate auditory fields in mice based on anatomical and functional parameters^1^. Tracing experiments in cats and mice confirmed parallel connections from the thalamus to both A1 and AAF^2,3^ and showed that in cats the vast majority of thalamocortical projections to A1 and AAF originate from largely non-overlapping cell groups of the medial geniculate body (MGB)^4^.

A1 and AAF are both tonotopically organized, but differ in the proportion of cortical area tuned to specific frequencies. In cats and ferrets, A1 neurons tuned to different frequencies are evenly distributed but AAF is characterized by an underrepresentation of cells tuned to mid-range frequencies^5–7^. Additionally, A1 neurons have narrower tuning, lower response thresholds, and decreased spontaneous activity in comparison to AAF neurons, consistently in many species^5,6,8^. On the other hand, AAF neurons show faster temporal modulation than A1 neurons^9^, significantly shorter receptive field durations and latencies^10^, better timing precision^11^, and fast-pass selectivity for the rate of frequency-modulated sweeps^12^. Despite this knowledge, it is still not clear whether their role in sound processing is distinct.

Using *in vivo* electrophysiological recordings in the mouse auditory cortex, we asked whether any tone properties are processed differently in these two primary auditory fields. Our results reveal significantly stronger responses to sound offset in AAF than in A1 neurons. Offset responses were reported previously in auditory cortex (ACx) neurons^13,14^ and their strength was compared between different auditory fields^15–17^ but their distinct properties in A1 and AAF were not described. Considering several inter stimulus intervals and sound durations, we show that offset is robust in AAF, but not in A1, and is positively correlated with sound duration. LFP analysis indicates that offset responses are not emergent in the cortex, but are of subcortical origin. Comparing response strengths between input (L4) and supragranular (L2/3) layers also suggest that offset responses are equally important in both layers in AAF, unlike in A1 where offsets in the input layers are dominating. Additionally, recordings from AAF neurons revealed that offset responses are poorly tuned and have weak tonotopy. Finally, we demonstrate that offset responses, even if significantly higher in fast spiking than in regular spiking neurons, are not specifically attributed to one of these two cell types. Our study reveals a crucial role of AAF in processing tone offsets, and thereby the possibility of distinct functions in sound processing for these two primary auditory cortices.

## Results

### Responses to sound onsets and offsets are distinct in A1 and AAF

**Figure 1:**
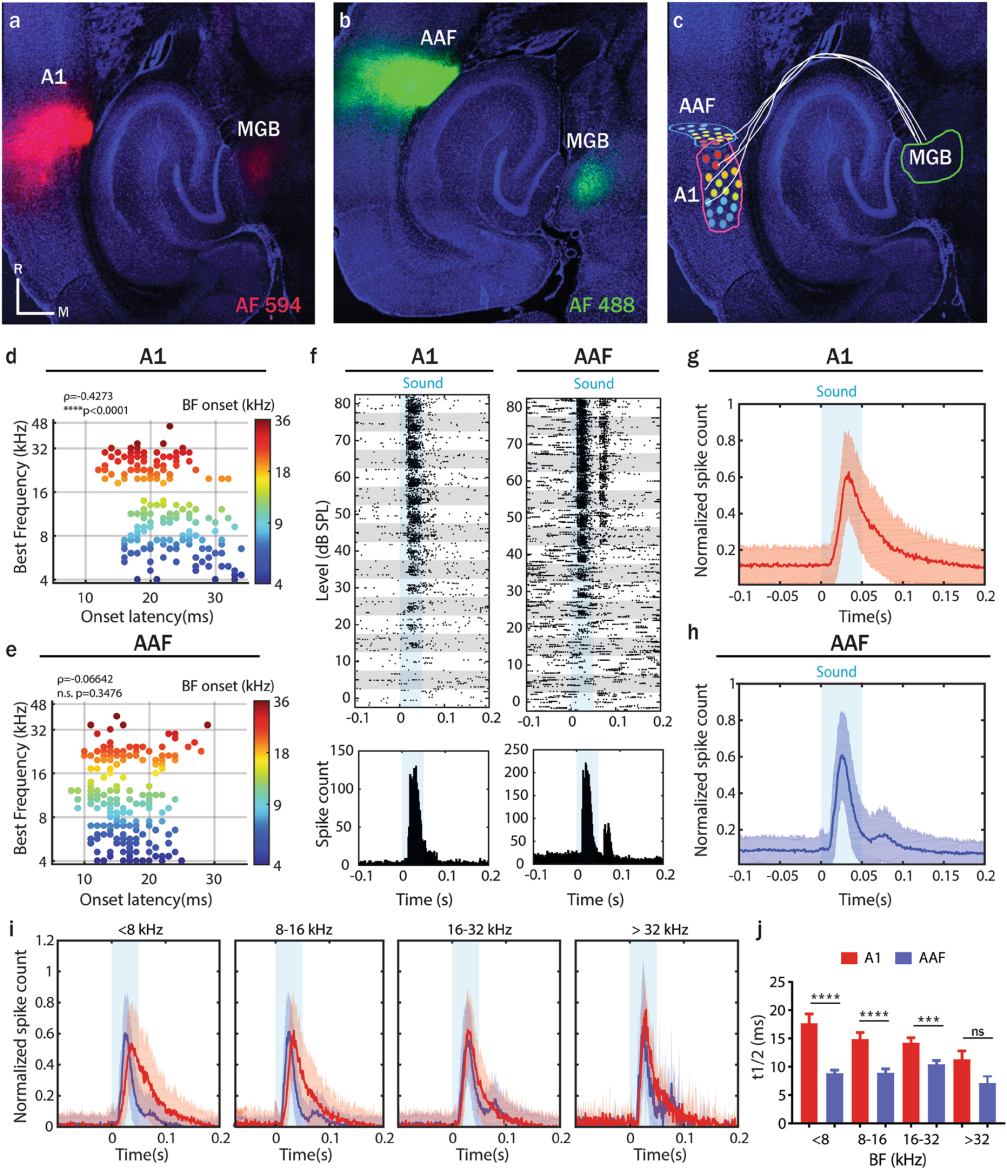
Responses to sound onsets and offsets are distinct in A1 and AAF. (**a**,**b**) Thalamocortical slice with CTB-red injected into A1, CTB-green injected into AAF and retrogradely labelled cells in MGB. (**c**) Thalamocortical slice with schematic indication of MGB, A1, AAF, their neural connections and tonotopy. The color code is as in (**d**). (**d**,**e**) Comparison of response latencies for differently tuned neurons in A1 and AAF. Latencies are color-coded to the neuron’s onset BF (correlation between onset latencies and BF: A1, ρ = −0.4273, p < 0.0001, n = 192; AAF, ρ = −0.0664, p = 0.3476, n = 202; Spearman correlation). (**f**) Raster plot and peristimulus time histogram (PSTH) of an example A1 (left) and AAF (right) neuron’s response to PTs (frequency varying between 4–48.5 kHz, sound level varying between 0–80 dB SPL, inter-stimulus-intervals equally distributed between 500 and 1000 ms). The blue shaded bar represents the tone presentation. (**g**,**h**) Average PSTH (mean ± SD) of A1 and AAF responses to PTs (same sound stimuli as in **f**). Panels (g–i) depict average responses collapsed across frequencies and sound levels. (**i**) Average PSTH (mean ± SD) of A1 (red) and AAF (blue) responses to PTs grouped according to their onset BF. (**j**) Comparison of half decay time (t1/2) of responses in A1 and AAF neurons grouped according to their onset BF. Time of half decay: A1: 17.67 ms (n = 39); 14.9 ms (n = 38); 14.26 ms (n = 63); 11.35 ms (n = 8); AAF: 8.88 ms (n = 71); 8.95 ms (n = 63); 10.42 ms (n = 55); 7.14 ms (n = 4) for neurons with BF < 8 kHz, 8–16 kHz, 16–32 kHz, >32 kHz respectively. Data represent mean ± SEM. ***p = 0.0003, ****p < 0.0001, ns p = 0.1535, Mann-Whitney test.

We first confirmed the primary nature of A1 and AAF by verifying that these two cortical regions receive parallel signals from MGB, combining *in vivo* multi-electrode electrophysiological recordings and retrograde tracing. Both regions were identified based on their functional tonotopy. Pure frequency tones (PTs) varying in frequency from 4 to 48.5 kHz and in level from 0 to 80 dB SPL presented with randomized inter-stimulus-intervals (ISIs) (500–1000 ms) were used to determine tuning receptive fields. By simultaneous recordings with 4 shank probes (200 µm distance between shanks in a 4 × 8 electrode configuration), we identified A1 and AAF based on their caudo-rostral and diagonal ventro-dorsal increase in best frequency (BF, defined as the frequency that elicited maximal response across all sound levels; see methods), respectively. We then injected retrograde tracers in both regions, identifying somas of neurons projecting to A1 or AAF. Confocal imaging of the thalamocortical slices revealed a robust signal in the MGB, confirming that both A1 (Fig. 1a) and AAF (Fig. 1b) receive signals from the auditory thalamus^2,3^. Moreover, this reinforced our identification of A1 and AAF through functional tonotopy, in respect to them being both primary cortices (Fig. 1c). In subsequent experiments, we therefore based the identification of A1 and AAF on functional tonotopy only.

We then compared sound evoked response patterns in A1 and AAF. Voltage traces across 32 channels were processed to extract single-unit cluster (SU) activity leading to 202 SU in AAF (7 animals) and 192 in A1 (6 animals). We confirmed that auditory responses have shorter onset latencies in AAF than in A1 neurons (Fig. 1d,e)^5,6,8,10^. In addition, our data revealed a gradient of response latencies in A1 (neurons tuned to high frequencies respond faster than neurons tuned to low frequencies) and no gradient in AAF (all neurons responded at similar time independently of their BF). Even more striking, responses to 50 ms long PTs revealed robust offset responses in AAF neurons, whereas less prominent offset was seen in A1 (Fig. 1f–h; Supplementary Fig. 1: Raster plot of Fig. 1f at higher resolution). By grouping A1 and AAF responses according to the neuron’s BF we saw an increase in the average firing rate following the end of the sound, confirming that offset responses in AAF are not frequency specific (Fig. 1i, increase in spike count following the end of the blue shadow (tone presentation)).

The analysis of the response decay in A1 and AAF (single exponential decay functions least-squares-fitted to PSTH of each neuron; Supplementary Fig. 2) allowed us to define another clear criteria to distinguish between A1 and AAF cells. Neurons in A1 showed significantly more sustained responses in comparison to AAF neurons (Fig. 1i,j). The lack of significance for neurons with BF higher than 32 kHz could be due to a lack of power (n = 8 and 4).

Response characteristics are to various degrees influenced by anaesthesia^8,18^. Therefore, we compared recordings in anesthetized and awake mice to establish whether offset responses were not induced by specific anesthetized state. We tested the same sound protocol as in the previous anesthetized experiment. We extracted 104 and 173 SU from awake recordings in A1 (5 animals) and AAF (6 animals), respectively. Similar to our results in anesthetized preparations, offset responses in awake mice were robust in AAF and weak in A1 (Supplementary Fig. 3). We also compared strength of offset responses between anesthetized and awake conditions. Spike rates at the offset were higher both in A1 and AAF for awake recordings, but this increase was larger and significant at a more stringent level in AAF neurons (Supplementary Fig. 4). This confirms the validity and relevance of our findings for awake and behaving conditions.

### Offset responses are significantly stronger in AAF

**Figure 2:**
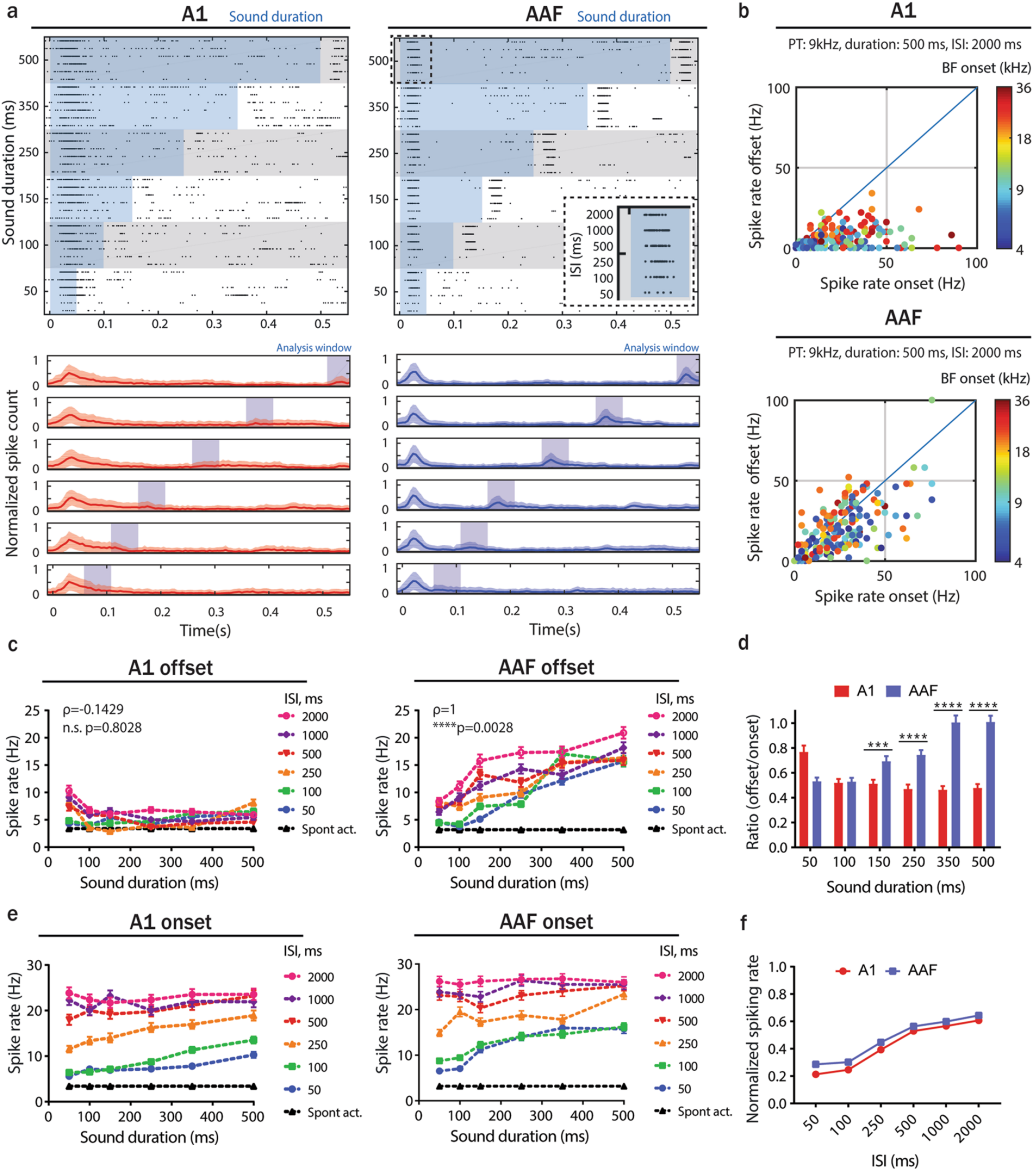
Offset responses are significantly stronger in AAF than in A1. (**a**) Raster plot of the example A1 (top-left) and AAF (top-right) neuron’s response to 9 kHz PTs played at 60 dB SPL with sound duration varying between 50 and 500 ms and ISI between 50 and 2000 ms and (bottom) PSTH (mean ± SD) averaged over all neurons population. The blue shaded bars represent the tone. (**b**) Comparison of strength of onset and offset responses in A1 and AAF for 500 ms 9 kHz PTs played with 2000 ms ISIs. Responses are color-coded to onset BF. (**c**) A1 (left) and AAF (right) offset responses to 9 kHz PTs with increasing duration across all ISIs’ (correlation between sound duration and response rate: A1, ρ = 0.1429, p = 0.8028, n = 191; AAF, ρ = 1, **p = 0.0028, n = 190, Spearman correlation). (**d**) Ratio of offset/onset responses in A1 and AAF for 9 kHz PTs with varying durations (50–500 ms) and 2000 ms ISI. Data represent mean ± SEM. ****p < 0.0001, ***p = 0.0007, Mann-Whitney test. (**e**) Onset responses of A1 (left) and AAF (right) neurons evoked by 9 kHz PTs of different durations (ISIs varied between 50 and 2000 ms). (**f**) Comparison of A1 and AAF onset responses to 9 kHz PTs (500 ms duration) with ISI varied between 50 and 2000 ms. There was no significant difference in forward suppression: p = 0.5887, Mann-Whitney test. Spike rates were normalized to maximum response of each individual neuron.

These robust offset responses in AAF may be a crucial indicator of distinct roles of A1 and AAF in sound duration encoding. Thus, we investigated whether offset responses in AAF were correlated to sound duration. We recorded responses to 60 dB SPL PTs (9 kHz) repeated 10 times with varying duration (50–500 ms) and inter-stimulus-intervals (50–2000 ms) (Fig. 2a). Based on responses to 500 ms tones played with 2000 ms ISI we found robust offset responses (higher than 2 standard deviations above the spontaneous activity) locked to the sound duration in 86.3% (164/190) of auditory responsive cells in AAF, but in only 40.3% (77/191) of A1 neurons (Fig. 2a,b). In A1 a cluster of cells tuned to high frequencies showed bigger offset responses than neurons with other BFs (Supplementary Fig. 5). This could be caused by recordings at the edge of A1/AAF, where neurons with high BF are located, or simply represent an offset responsive neuron cluster in A1. We also found that offset responses in AAF increase with sound duration, unlike in A1 (Fig. 2c). The strength of offset relative to onset responses was significantly higher in AAF than in A1 for sounds with longer duration (Fig. 2c,d). The high offset responses reported for the shortest sounds (50 ms) in A1 neurons result from the overlap between onset and offset responses with these short sounds (Fig. 2c,d).

We then estimated the forward suppression that could potentially be caused by offset responses based on the relationship between onset response amplitudes and ISIs. There was no significant difference in forward suppression between A1 and AAF neurons (Fig. 2e,f). We additionally analysed the correlation between offset and onset responses at each ISI (Supplementary Fig. 6). We found a positive correlation between offset and onset response in both A1 and AAF, demonstrating that the stronger offsets do not influence a following onset response, even when the time between the end of a tone and the start of the next one is short. These results are another indication that onset and offset responses might be driven by non-overlapping sets of synapses^19^.

### Robust offset responses in AAF are not unique to pure frequency tone stimulations

**Figure 3:**
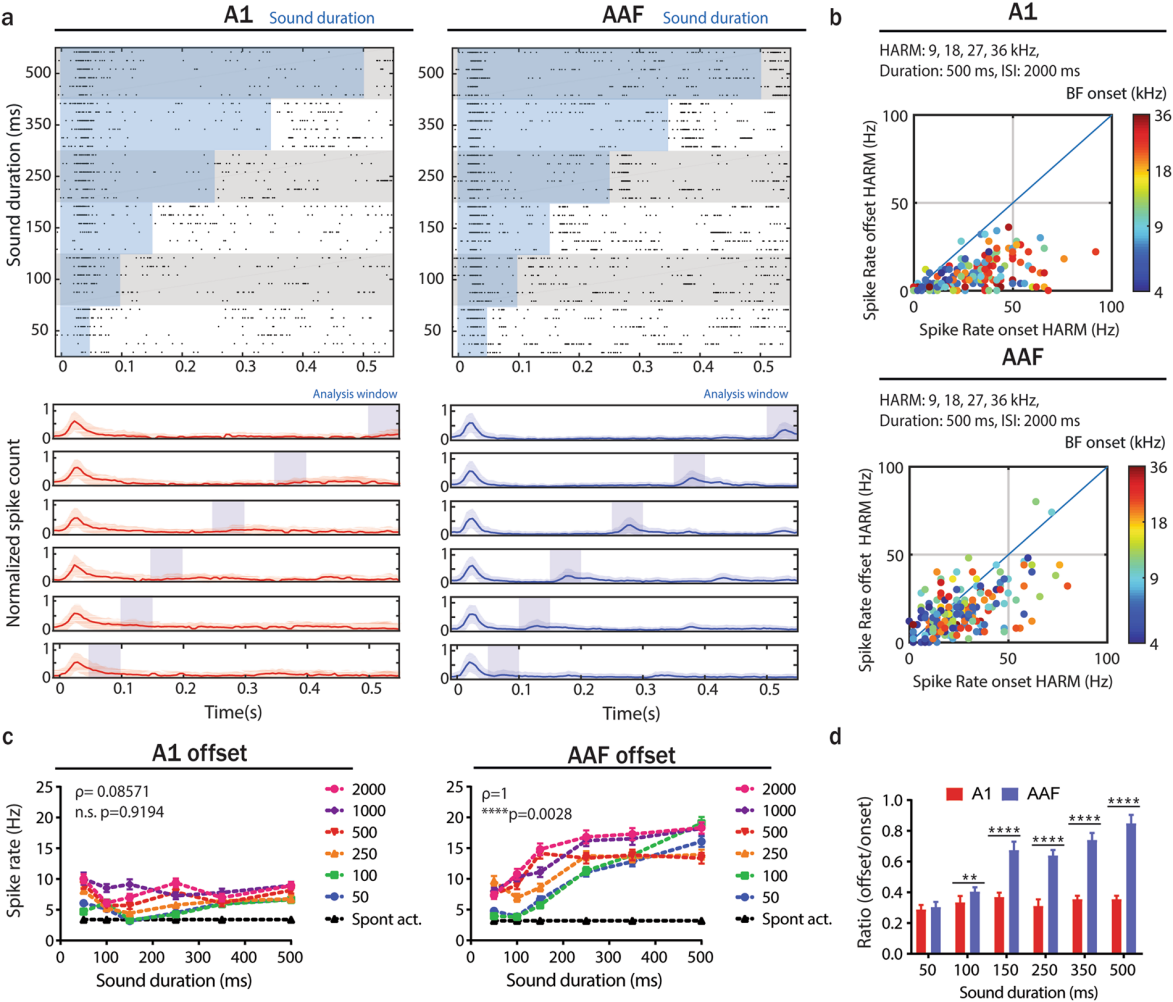
Offset responses to harmonic tone stimulation are more robust in AAF than in A1. (**a**) Raster plot of an example A1 (top-left) and AAF (top-right) neuron’s response to HTs (9 + 18 + 27 + 36 kHz) played at 60 dB SPL with sound duration varying between 50 and 500 ms and ISI between 50 and 2000 ms and (bottom) PSTH (mean ± SD) averaged over all neurons. The blue shaded bars represent the tone presentation. The example neurons do not correspond to those presented in Fig. 2a. (**b**) Comparison of strength of onset and offset responses in A1 and AAF neurons for 500 ms HTs (9 + 18 + 27 + 36 kHz) played with 2000 ms ISIs. Responses are color-coded to the onset BFs. (**c**) A1 (left) and AAF (right) offset responses to HTs (9 + 18 + 27 + 36 kHz) with increasing duration across all ISIs’ (correlation between sound duration and response rate: A1, ρ = 0.0857, p = 0.9194, n = 191; AAF, ρ = 1, p = 0.0028, n = 190, Spearman correlation). (**d**) Ratio of offset/onset responses in A1 and AAF for HTs (9 + 18 + 27 + 36 kHz) with varying durations (50–500 ms) and 2000 ms ISI. Data represent mean ± SEM. ****p < 0.0001, **p = 0.0031, Mann-Whitney test.

In the previous experiments we used PTs of different frequencies, levels and durations to investigate the presence of offset responses. To investigate whether offset responses were specific to PTs stimulation we performed the same experiment with harmonic tones (HTs) instead of PTs. HTs consisted of four frequency components: f_0_, f_1_, f_2_ and f_3_, where f_1_ = 2*f_0_; f_2_ = 3*f_0_; f_3_ = 4*f_0_. We recorded responses to 60 dB SPL HTs (9, 18, 27, 36 kHz) with varying duration (50–500 ms) and ISIs (50–2000 ms) (Fig. 3a,b). Based on responses to 500 ms HTs played with 2000 ms ISI we found robust offset responses in 88.4% of auditory responsive cells in AAF and in 61.8% in A1. As for PT, we found offset responses increasing with sound duration in AAF but not in A1 (Fig. 3c,d). Comparing responses to PTs and HTs demonstrated that the strength of offset relative to onset responses was significantly higher for HTs than for PTs (Supplementary Fig. 7). In AAF HTs did not cause any significant change in offset/onset ratio compared to PTs. Therefore, although the number of A1 cells with significant offset responses to HTs was increased in comparison to PTs, the strength of offset relative to onset responses was again significantly higher in AAF than in A1 for all sound durations longer than 50 ms (Fig. 3d). These results lead to the conclusion that predominant offset responses in AAF are not unique to PT stimulations.

### Offset responses do not emerge in the cortex

**Figure 4:**
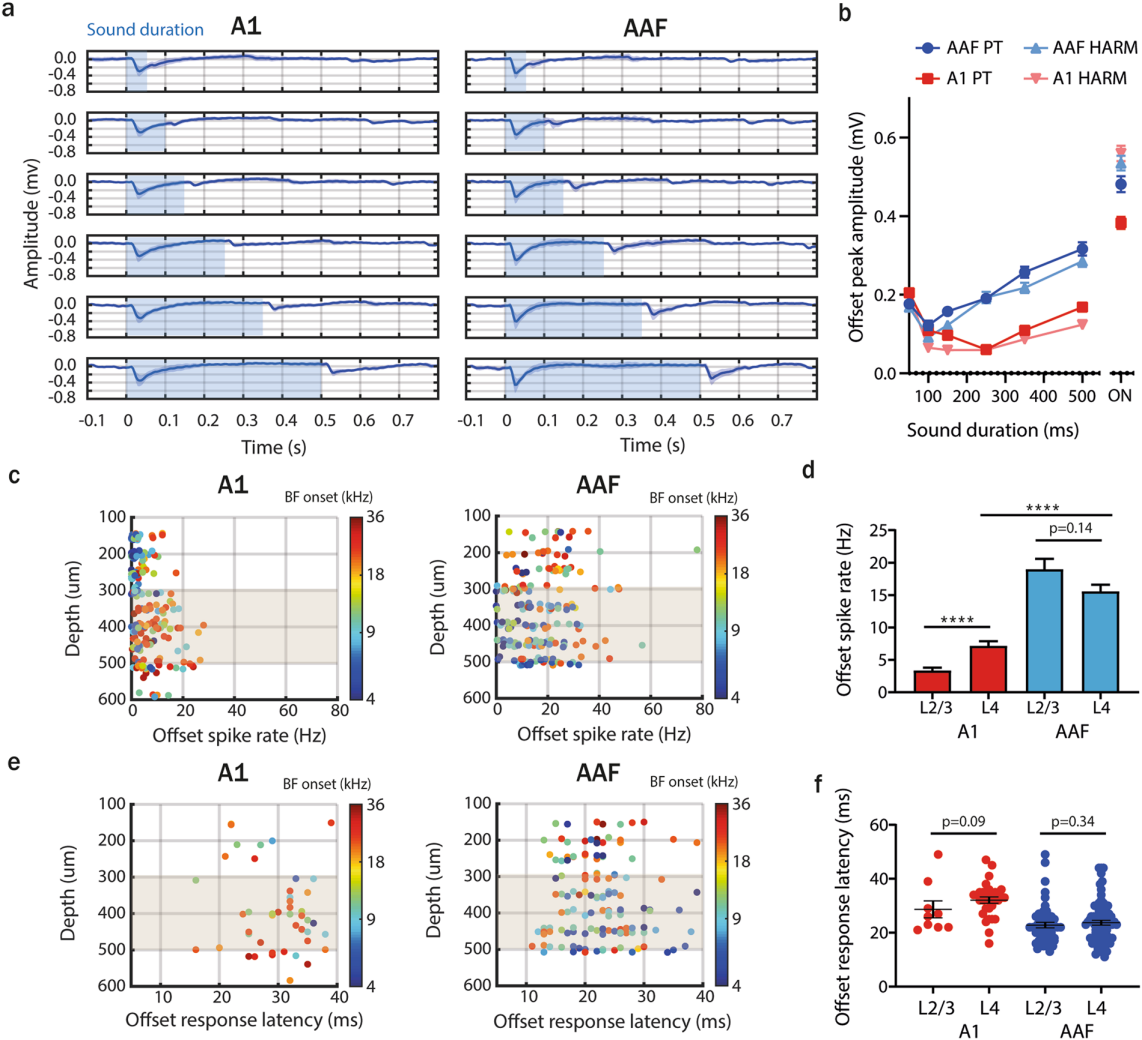
Offset responses do not emerge in the cortex. (**a**) Averaged LFP signal (mean ± SD) from L4 A1 (n = 6 animals, 72 recording sites) and AAF (n = 7 animals, 84 recording sites) neurons in response to 9 kHz PTs played at 60 dB SPL with sound duration varying between 50 and 500 ms and ISI between 50 and 2000 ms. The blue shaded bars represent the tone. (**b**) Comparison of onset and offset peak amplitude of LFP signal in L4 A1 and AAF neurons in response to PTs and HTs with different durations. Data represent mean ± SEM. (**c**) Offset spike rate (evoked by 9 kHz PTs, duration: 500 ms) in A1 (left) and AAF (right) neurons as a function of recording site depth. Responses are color-coded to the neuron’s onset BF. The gray shaded bars represent L4. (**d**) Comparison of offset spike rate in L2/3 and L4 of A1 and AAF neurons. Data represent mean ± SEM. (A1: ****p < 0.0001, L2/3 n = 72; L4 n = 88; AAF: p = 0.14, L2/3 n = 69; L4 n = 128, Mann-Whitney test, L4: ****p < 0.0001, A1: n = 88, AAF: n = 128) (**e**) Offset response latency in A1 (left) and AAF (right) neurons as a function of recording site depth. Responses are color-coded to the neuron’s onset BF. The gray shaded bars represent L4. (**f**) Comparison of offset spike rate in L2/3 and L4 of A1 and AAF neurons. Data represent means and individual values. (A1: p = 0.09, L2/3 n = 9; L4 n = 31; AAF: p = 0.34, L2/3 n = 52; L4 n = 76, Mann-Whitney test).

We next performed LFP analysis of our recordings in the thalamorecipient layer to address whether offset responses are an emergent property of the auditory cortex. Our recordings span the range of 150 to 600 µm from the pia surface, corresponding mainly to L2/3 (150–300 µm) and L4 (300–500 µm)^20^; we only analysed data recorded in L4 here. Based on the protocols with varied sound duration and intervals we could see, as for the spike rate analysis (Fig. 2), that offset responses in AAF were more robust than in A1 for both PTs (9 kHz) and HTs (9 + 18 + 27 + 36 kHz; Fig. 4a,b, Supplementary Fig. 8). LFP peak amplitude at the sound offset increased together with sound duration (Fig. 4b) as previously shown with spike analysis (Fig. 2c, right panel). The presence of offset responses within the L4 A1 and AAF LFP signals suggests that offset responses are likely to be inherited from subcortical nuclei rather than only arising in primary auditory regions.

By analysing offset responses as a function of recording depth, we show that offset responses are of similar amplitude in all recorded depth in AAF, whereas A1 displays significantly stronger responses in L4 than in L2/3 (Fig. 4c,d). Lack of cortical processing could result in a lower spike rate in a feedforward L4 to L2/3 circuit^20^. This suggests that, in addition to receiving offset responses from subcortical nuclei in the input layer (L4), offset responses are further processed in supragranular layers (L2/3) of AAF, but less so in A1. An analysis of latencies indicates that AAF offset responses appear equally fast in L4 and L2/3 (p = 0.34, Mann Whitney Test; Fig. 4e,f). Interestingly in A1, offset responses appear slightly faster in L2/3 than in L4 (p = 0.09), suggesting that A1 L2/3 offset responses could possibly be of a different origin than the input layer. Altogether, these results indicate that the differences between A1 and AAF offset processing could have two origins. First, AAF receives stronger offset in the input layer than A1. Second, intracortical processing of offset responses in L2/3 is stronger in AAF than in A1.

### Offset responses in AAF have weak tonotopy, irregular tuning and are present in both fast and regular spiking neurons

**Figure 5:**
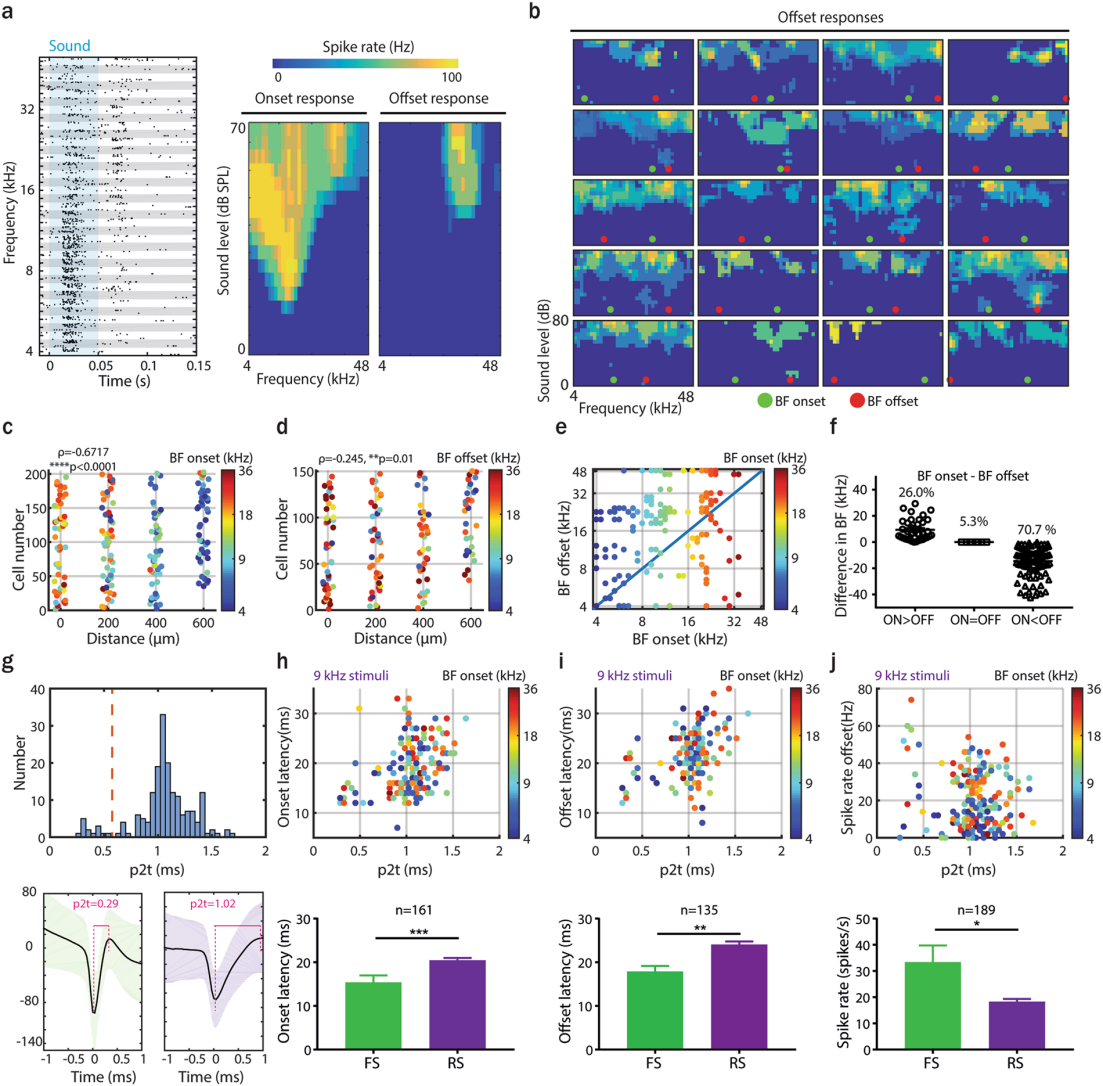
Offset responses in AAF have weak tonotopy, irregular tuning and are present in both fast and regular spiking neurons. (**a**) Example of distinct tuning receptive fields (TRF) of onset and offset responses of an AAF neuron. (**b**) Examples of offset TRF of AAF neurons with indication of onset (green) and offset (red) BF. (**c**,**d**) Comparison of onset and offset BF of AAF neurons, displayed as a relative distance between electrode shafts. Responses are color-coded to onset (**c**) or offset (**d**) BF (correlation between BF and relative distance between electrode’s shaft: onset, ρ = 0.6717, ****p < 0.0001, n = 202; offset, ρ = 0.245, **p = 0.01, n = 150, Spearman correlation). (**e**,**f**) Comparison of onset and offset BF for AAF neurons (responses are color-coded to the neuron’s onset BF). (**g**) Distribution of peak-to-trough times (p2t) of AAF neurons. The red line defines the middle of bimodal distribution (p2t = 0.575). Example of fast (p2t = 0.29; bottom left) and regular (p2t = 1.02; bottom right) spiking unit waveforms. (**h**) Comparison of onset latency of onset responses as a function of p2t of spike waveforms to 9 kHz PT (top). Responses are color-coded to onset BF. Corresponding average values for FS and RS neurons (bottom). Data represent mean ± SEM. ***p = 0.0003, Mann-Whitney test. (**i**) Comparison of response onset latency of offset responses as a function of p2t of spike waveforms (top). Corresponding average values for FS and RS neurons (bottom). Data represent mean ± SEM. **p = 0.0019, Mann-Whitney test. (**j**) Comparison of offset response spike rates as a function of p2t of spike waveforms (top). Corresponding average values for FS and RS neurons (bottom). Data represent mean ± SEM. *p = 0.02, Mann-Whitney test.

Because of their predominance in AAF, we then characterized offset as a function of onset responses in this primary cortical region. First, we compared onset and offset tuning receptive fields (TRF) for all recorded AAF neurons. We found a few neurons with clear onset and offset tuning (Fig. 5a). Most other cells revealed very broad, irregular and poorly tuned offset receptive fields (Fig. 5b,c). By aligning onset and offset BFs of the recorded neurons according to the electrode shafts, our analysis revealed a robust tonotopy for onset (Fig. 5c), but weaker tonotopy for offset responses (Fig. 5d). Significant tonotopy for onset was also confirmed for A1 (Supplementary Fig. 9; ρ = 0.719, ****p < 0.0001, Spearman correlation). 70.7% of offset responses were tuned to higher frequencies than onset responses, 26.0% to lower frequencies, and 5.3% showed the same BF for onset and offset responses (Fig. 5e,f; tolerance: 0.1 octave).

We then asked whether different cell types play distinct roles in onset and offset responses. We distinguished fast (FS) and regular (RS) spiking neurons based on their spike waveforms, and analysed their responses to 9 kHz PTs played at 60 dB SPL for 500 ms. The peak-to-trough times (p2t) of our sorted units gave a bimodal distribution (Fig. 5g; bin size = 0.05 ms). FS units were defined as having a p2t smaller than the minimum between the two peaks of the p2t distribution (0.575 ms), in accordance with previous studies^21^. FS units had significantly shorter onset (Fig. 5h, FS: 15.42 ± 1.57, n = 12 RS: 20.47 ± 0.51, n = 149 cells) and offset (Fig. 5i, FS: 17.91 ± 1.25, n = 11, RS: 24.09 ± 0.67, n = 124 cells) response latencies than RS cells, indicating that FS neurons can play an important role in processing sound onset as well as offset. However, even if significantly stronger in FS than RS cells, offset responses were highly robust in both cell populations (Fig. 5j, FS: 33.43 ± 6.33, n = 14 cells, RS: 18.33 ± 1.03, n = 175 cells), suggesting that offset responses are not specifically attributed to one of these two cell types.

## Discussion

Distinguishing responses to sounds in different auditory cortical regions is crucial to understand how auditory signals are processed at the cortical level. Here we reveal for the first time a higher percentage of neurons responding to sound offset in AAF in comparison to A1. Our observations were drawn from the analysis of neuronal response properties in both anesthetized and awake conditions, with pure or harmonic tones as the auditory stimuli. Our results also reveal that offset responses are equally represented in both input and supragranular layers in AAF, but not in A1 where offsets in the input layers are dominating. Finally, we demonstrate that offset responses in AAF, even if significantly higher in FS than in RS neurons, are not specifically attributed to one of these two cell types. Altogether, our results indicate that both subcortical input and intracortical processing differences contribute to the distinct processing of sound offsets in A1 and AAF.

The high number of offset responses in AAF (more than 85%) has not been previously reported. In general, there has been a significant asymmetry in the relevance of excitatory onset and offset responses in auditory processing. Previous studies reported 25–75% of offset responsive cells^16,22–24^ or even absence of them in A1^25^, but none reported their prevalence in AAF. The reason behind the fact that the prevalence of offset responses in AAF compared to A1 was not reported before may be manifold. First, most previous studies aimed at investigating offset responses focused on A1, whereas AAF – identified by our study to be crucial for offset, was often neglected. Second, most studies comparing offset responses among auditory fields were performed with imaging techniques, which limits recordings to superficial layers only^15,17^. Then, offset responses are highly influenced by duration (Fig. 2c), intensity as well as rise-fall time of the sound^15,19,24^, raising the possibility that AAF offset responses could not be clearly seen due to the characteristics of the stimuli used in previous studies. Finally, the use of pentobarbital as anaesthetic might explain the lack of offset responses in some studies. Our anesthetized recordings under ketamine confirm that this anaesthetic does not prevent offset response occurrence (Fig. 1f,g).

Auditory responses are shaped by stimulus characteristics. For example, our data reflect an increase in the percentage of offset responses evoked in A1 neurons by HTs as compared to PTs. This substantial increase was not observed in AAF. HTs evoke bigger auditory responses than PTs in general, and they could therefore contribute to an increased number of offset cells in A1. The lack of increase in AAF could be explained by the fact that the percentage of offset responses to PTs is already high in this brain region. Another possibility could be that A1 and AAF display different amount of offset responses for distinct stimuli. A broader group of sounds should be tested to fully address this question.

We found offset responses in more than 40% of A1 and 80% of AAF neurons. These percentages lead us to expect a bigger involvement of AAF in offset processing, but we cannot exclude a potential functional importance of A1 for the processing of sound termination. In general, it is known that the proportion of offset cells increase in awake animals, especially when animals are engaged in a behavioural task. For example, offset responses were found in 90% of A1 neurons in awake monkeys attending to tones^26^. In our study, we measure an increase of offset response strength from anesthetized to awake conditions in A1, but this increase is even higher in AAF (Supplementary Fig. 4), confirming again this field’s importance for offset processing.

Within AAF, onset and offset responses also encode auditory information differently. Their distinct tuning fields (Fig. 5a,b), different tonotopy (Fig. 5c,d)^17^ and lack of offset-onset forward suppression (Fig. 2f, Supplementary Fig. 6) suggest that they could be generated at distinct locations earlier in the auditory pathway^19,27,28^, and then processed independently.

The predominance of offset responses in AAF and their significantly smaller amount in A1 (Fig. 2), the faster and more transient responses in AAF than in A1 (Fig. 1e,i,j) as well as the dependence of response strength on tone duration in AAF (Fig. 2c,d) reveal intriguing differences between these two primary auditory cortices. What could be the underlying mechanisms leading to such differences? Previous studies indicate that the MGB, the nucleus preceding A1 and AAF in the auditory pathway, contains offset responsive cells^29,30^. Our LFP and layer specific analyses confirm that offset responses do not emerge in the auditory cortex but that they are received from subcortical nuclei in the input layer (L4) (Fig. 4). These responses are however significantly stronger in AAF than in A1. In addition, the robust offset responses in L2/3 suggest that AAF (Fig. 4c,d) processes offsets further. On the contrary, A1 does not have as strong responses in supragranular layers. These results indicate that the difference in sound offset processing between A1 and AAF are both of subcortical and intracortical origins. Interestingly, A1 L2/3 offset responses appear faster than L4 responses (Fig. 4e,f). Could it be that A1 L2/3 offset responses originate from AAF offset responses, as these two regions are highly interconnected^7,31^? Further studies will have to be performed to fully address the mechanisms behind the distinct sound processing in A1 and AAF.

Intracortically, both fast and regular spiking neurons are involved in processing information on sound onset and offset (Fig. 5g–j). One could expect FS interneurons to be more involved in offset encoding as they can provide dynamic gain control of neural activity^32^. Our results indeed reveal higher offset responses in FS than RS cells (Fig. 5j). However, as offset responses are robust in both cell types, they cannot be specifically attributed to one of them. Moreover, the involvement of FS neurons is similar for onset and offset responses: they have shorter latencies and stronger firing rates than RS neurons, as also indicated in the awake mouse auditory cortex^32^. Thus the stronger offset responses and faster latencies reflect a general principle of the role of this class of inhibitory neurons in cortical processing, where they control and modulate local circuit processing.

With its robust offset responses, transient onset responses and shorter onset latencies, AAF is likely to have a more prominent role for encoding sound timing^33^, and, by extension, for all time-varying signals so important in vocalization. Functionally, offset responses might be important to perceive sound termination, to detect gaps in an otherwise continuous sound, or to detect changes in amplitude modulated signals^34^. Previous studies showed that the auditory cortex is required for the detection of short gaps (<75 ms) but not for the detection of long gaps (>100 ms)^35^. Our data display offset responses predominantly for longer duration signals, or for shorter signals with longer ISIs (Fig. 2c). This reflects a possible particular role of AAF offsets in processing slower transients. The question of the potential role of AAF offset response in gap detection therefore remains open. One could speculate that the overlap of onset and offset response we see in A1 for short tones (Figs 1f and 2a) is contributing to short gap sensitivity, and that AAF would play a more important role for the detection of longer gaps.

Taken together, our findings reveal distinct sound processing in the mouse two primary auditory cortices. The different responses to sound termination in A1 and AAF emphasizes the complexity of sound processing at the cortical level. Our data also indicate that A1 and AAF should be clearly defined and distinguished during experiments as they represent sound parameters differently. But why does the auditory cortex need two primary regions? One can speculate that auditory stimuli, as compared to other sensory stimuli, have more complex and challenging temporal characteristics. We believe that the distinct roles of A1 and AAF are likely to be used complementarily as there is considerable overlap in A1 and AAF response properties. If and how other sound features are differentially encoded in A1 and AAF remains to be elucidated.

## Methods

### Surgical procedures

All experimental procedures were carried out in accordance to Basel University animal care and use guidelines, and were approved by the Veterinary office of the Canton Basel-Stadt, Switzerland. They were performed on adult (7–9 weeks) male or female C57BL/6 J mice (Janvier, France).

Mice were anesthetized with intraperitoneal injection of ketamine/xylazine (80 mg/kg and 16 mg/kg, respectively) and subcutaneous injection of bupivacain/lidocain (0.01 mg/animal and 0.04 mg/animal, respectively) was used for analgesia. Ketamine (45 mg/kg) was supplemented during surgery as needed. For surgery, mice were head fixed and their body temperature was kept at 37 °C with a heating pad (FHC, ME, USA). Craniotomy (~2 × 2 mm^2^) was performed with a scalpel just above the right auditory cortex and covered with silicone oil. For awake recordings, the surgery was performed under isoflurane (4% for induction, 1.5 to 2.5% for maintenance) and the brain was additionally covered with a silicone casting compound (Kwik cast, World Precision Instruments, Inc. FL, USA) during the 2 h recovery period from the anaesthesia.

### Recordings

The electrophysiological recordings were performed in anesthetized mice (A1: n = 6; 4 females: 36, 45, 39, 23 cells; 2 males: 28, 21 cells; AAF: n = 7; 3 females: 27, 45, 25 cells; 4 males: 32, 26, 31, 16 cells) except for the data presented in Supplementary Fig. 3 and 4 which were acquired in awake experiments (A1: n = 5; 5 females: 16, 26, 23, 22, 17 cells; AAF: n = 6; 5 females: 22, 9, 33, 41, 40 cells; 1 male: 28 cells). Mice were head fixed and placed on a heating pad (or in the cardboard tube for awake recordings) inside a sound box. The body temperature was kept at 37 °C. Extracellular recordings were conducted in A1 and AAF, which were identified based on the functional tonotopy (caudal-rostral increase in BF for A1, and ventro-dorsal increase in BF for AAF). Multi-channel extracellular electrodes with 32 channels (A4 × 8-5 mm-50-200-177-A32, Neuronexus, MI, USA) were inserted orthogonal to the brain surface with a motorized stereotaxic micromanipulator (DMA-1511, Narishige, Japan) at a constant depth (tip of electrode at 575 ± 25 µm from pia). Responses from extracellular recordings were digitized with a 32-channel recording system (RZ5 Bioamp processor, Tucker Davis Technologies, FL, USA) at 24414 Hz. Single unit clusters were identified from raw voltage traces using kilosort (CortexLab, UCL, London, England) followed by manual corrections based on the inter-spike-interval histogram and the consistency of the spike waveform (phy, CortexLab, UCL, London, England) and further analysed in MATLAB (Mathworks, MA, USA). Neural activity was considered as auditory responsive when it exceeded twice the standard deviation of the spontaneous activity. Local Field Potential signal was obtained by applying low pass-filtering with a cut-off 100 Hz on the raw voltage traces.

### Auditory stimulation

Sounds were generated with a digital signal processor (RZ6, Tucker Davis Technologies, FL, USA) at 200 kHz sampling rate and played through a calibrated MF1 speaker (Tucker Davis Technologies. FL, USA) positioned at 10 cm from the mouse’s left ear. Stimuli were calibrated with a wide-band ultrasonic acoustic sensor (Model 378C01, PCB Piezotronics, NY, USA).

#### Tuning receptive fields

To determine BF and tuning receptive fields we used PTs (50 ms duration, randomized ISI distributed equally between 500 and 1000 ms, 2 repetitions, 4 ms cosine on and 0.01 ms cosine off ramps) varying in frequency from 4 to 48.5 kHz in 0.1 octave increments and in level from 0 to 80 dB SPL in 5 dB increments. Tuning receptive fields, best frequency and spiking rates were calculated in fixed time windows: A1 onset: 10–60 ms, A1 offset: 60–110 ms, AAF onset: 8 58 ms, AAF offset: 58–108 ms. TRFs were smoothed with a median filter (4 × 4 sampling window) and thresholded to 0.2 of peak amplitude. d’, a parameter used to assess the tuning quality of the TRF, was calculated as the difference in mean spike count within the tuning field and mean spike count outside the boundary of tuning field divided by their arithmetic average standard deviation (modified method from^8^). For all figures we used units with onset response tuning quality higher than 1 (d’ > 1). For Fig. 5d,e we also assessed offset response tuning quality and analysed cells with d’ > 0.5. Onset and offset BF was defined as the frequency that elicited maximal response across all sound levels. Onset and offset latency was determined as the first time point in which the smooth PSTH (kernel = hann(9)) collapsed across all tested stimuli exceeded with 2 standard deviations the spontaneous activity (binning size: 1 ms). In Fig. 1 onset response latency was calculated based on the protocol used to determine tuning receptive fields (PTs, 50 ms duration, randomized ISI distributed equally between 500 to 1000 ms, 2 repetitions, 4 ms cosine on and 0.01 ms cosine off ramps; varying in frequency from 4 to 48.5 kHz in 0.1 octave increments and in level from 0 to 80 dB SPL in 5 dB increments). In Figs 4e,f and 5 onset and offset response latencies were calculated based on the responses to 9 kHz PTs of 500 ms presented with ISIs of 250, 500, 1000 or 2000 ms at 60 dB SPL, 10 repetitions, 4 ms cosine on and 0.01 ms cosine off ramps. The number of cells presented in Fig. 5h–j differ because for some cells onset or offset response latency could not be assessed if the smoothed neuron’s onset or offset response was lower than twice the spontaneous activity standard deviation. Single exponential decay functions were least-squares-fitted to PSTH of each neuron’s onset response. Exponential fit was accepted if the explained variation (n2)

$$ExplainedVariation=1-\frac{\sum {({\rm{ObservedResp}}-{\rm{predictedResp}})}^{2}}{\sum {\rm{ObservedResp}}-{\rm{mean}}({\rm{ObservedResp}}){)}^{2}}$$

was bigger than 0.7.

#### Tone duration responses

To study responses to tones with different durations, we used 10 repetitions of PTs (9 kHz) or HTs (9 + 18 + 27 + 36 kHz) with 4 ms cosine on and 0.01 ms cosine off ramps, which were varied in duration (50, 100, 150, 250, 350, 500 ms), ISI (gap between 2 stimuli of 50, 100, 250, 500, 1000, 2000 ms) and played at 60 dB SPL. Sound level ratio (in V) of harmonic components was as follow: 1f_0_:1/2f_1_:1/4f_2_:1/8f_3_. For this protocol, f_0_ was fixed to 9 kHz because of (1) irregular and poorly tuned offset receptive fields found in AAF, which did not allow to designate offset BF with high accuracy and therefore any analysis of responses as a function of offset BF would be imprecise, (2) the fact that 9 kHz PTs evoked significant offset responses in almost all tested AAF but not A1 neurons irrespectively of their tuning (Supplementary Fig. 5), (3) the wish to test harmonic stimuli with f_0_ to f_3_ within the range of our stimulation parameters.

### Retrograde tracers

Retrograde tracing was performed as previously described^36^. Briefly, mice were anesthetized with ketamine/xylazine (80 mg/kg). Following the initial microelectrode mapping, retrograde tracers, cholera toxin beta subunit (CTB) conjugated to either 594 (red, Invitrogen cat# 34777) or Alexa 488 (green, Invitrogen cat# 34775) were injected into A1 or AAF, (N = 2 for each region). After injections, the surgical area was closed and the animal allowed to recover for approximately 96 hours before transcardial perfusion. Animals were first perfused with 0.9% saline and then with a cold (4 °C) solution of 4% paraformaldehyde prepared in 0.1 M phosphate buffer (pH 7.4). Following perfusion, the brains were removed and placed in 30% sucrose for 1 to 3 days. Blocks containing the auditory cortex were cut with a vibratome in a modified horizontal plane that included A1 and MGB. Slices (80 µm) were visualized with a confocal laser-scanning microscope (Zeiss LSM700) with a Plan-apochromat 10×/0.8 NA objective.

### Statistical analysis

Statistical tests were performed with GraphPad Prism software version 7.03 (GraphPad Software, USA). The standard error of the mean was calculated to quantify the amount of variation between responses from different populations. Nonparametric, unpaired Mann-Whitney test was used to calculate whether there were any significant differences between medians of recordings in A1 and AAF. Wilcoxon paired test or paired t-test were used to compare differences between paired values obtained in different treatments which have (paired t-test) or not have (Wilcoxon test) Gaussian distribution. Spearman correlation tests were used to test for significant associations between pairs of variables measured with rankings.

## Supplementary information

Supplementary Information

## Data Availability

Data are available upon reasonable request.
