# Supplementary material for: Distinct processing of tone offset in two primary auditory cortices
Source: Sci Rep. 2019 Jul 3;9:9581. doi: 10.1038/s41598-019-45952-z (PMC6610078; doi:10.1038/s41598-019-45952-z)
Supplement: Supplementary file 1 — Supplementary Information [file 41598_2019_45952_MOESM1_ESM.pdf]

Distinct processing of tone offset in two primary auditory cortices

Magdalena Sołyga [1] and Tania Rinaldi Barkat [1, *]

[1]Brain & Sound Lab, Department of Biomedicine, Basel University, 4056 Basel, Switzerland
* tania.barkat@unibas.ch

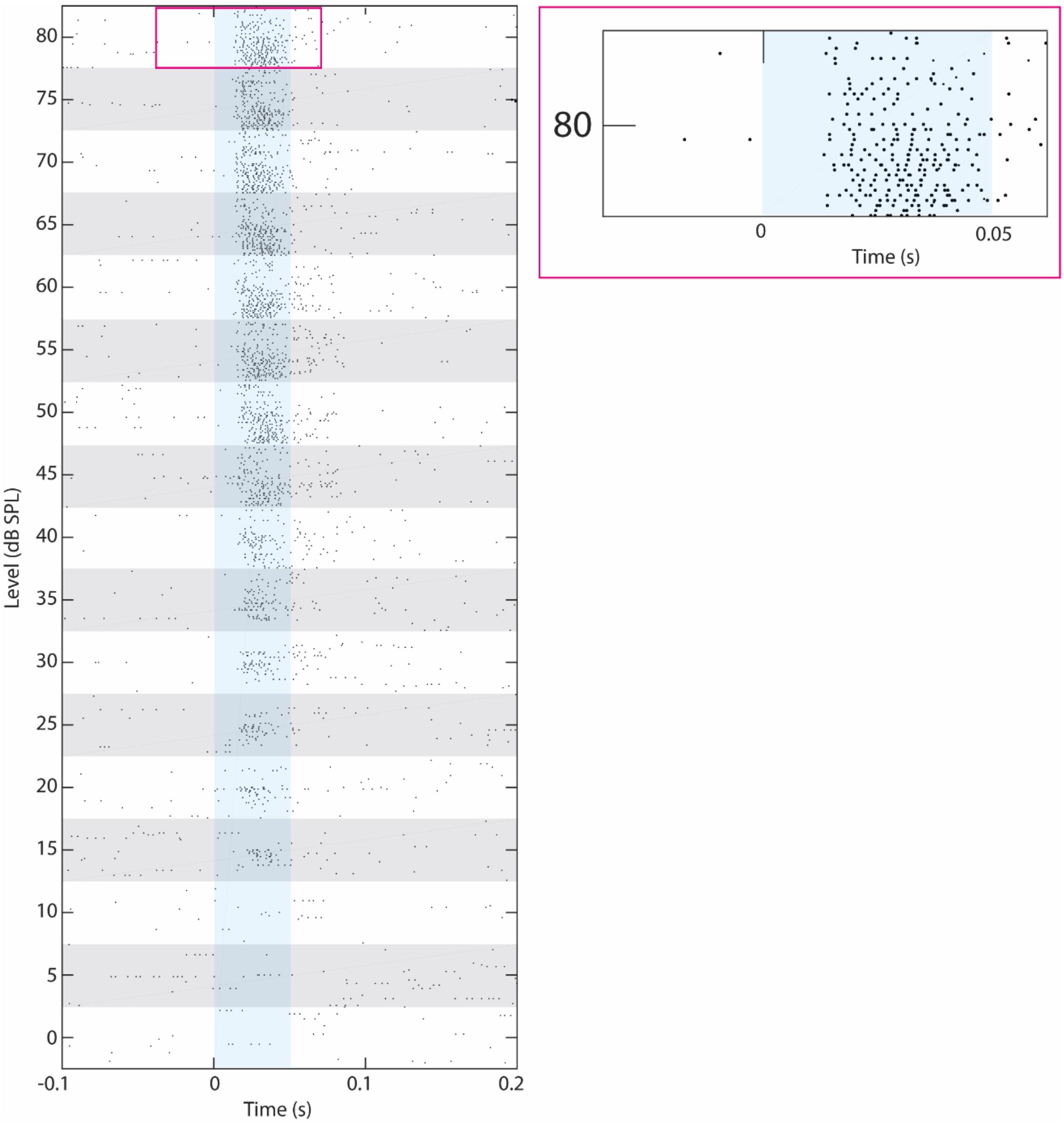

Supplementary Figure 1 Raster plot of Fig. 1f (left) at higher resolution. Each line of the raster plot represents spikes evoked by two repetitions of a tone at one frequency and one sound level. 37 frequencies (varying between 4 - 48.5 kHz in 0.1 octave increments) were tested for each sound level. The blue shaded bar represents the tone presentation.

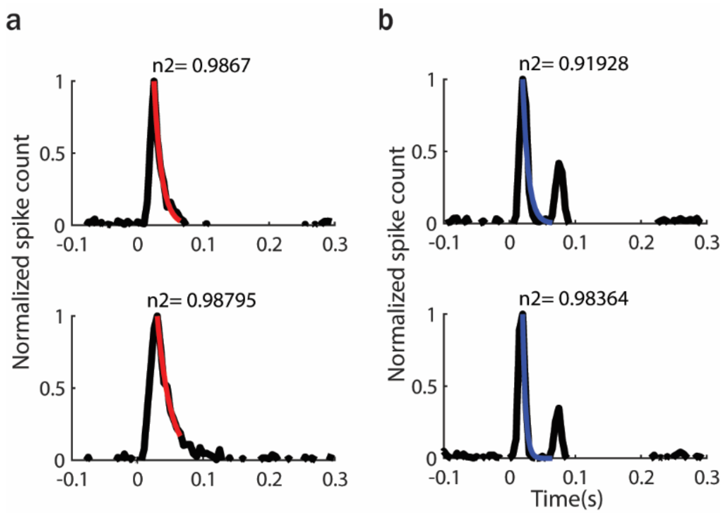

Supplementary Figure 2 Exponential decay model used to assess sustainability of neuron's response. (**a, b**) Example fit of exponential decay model to PSTH of 2 A1 (**a**) and 2 AAF (**b**) neurons' responses (n2: amount of variance explained).

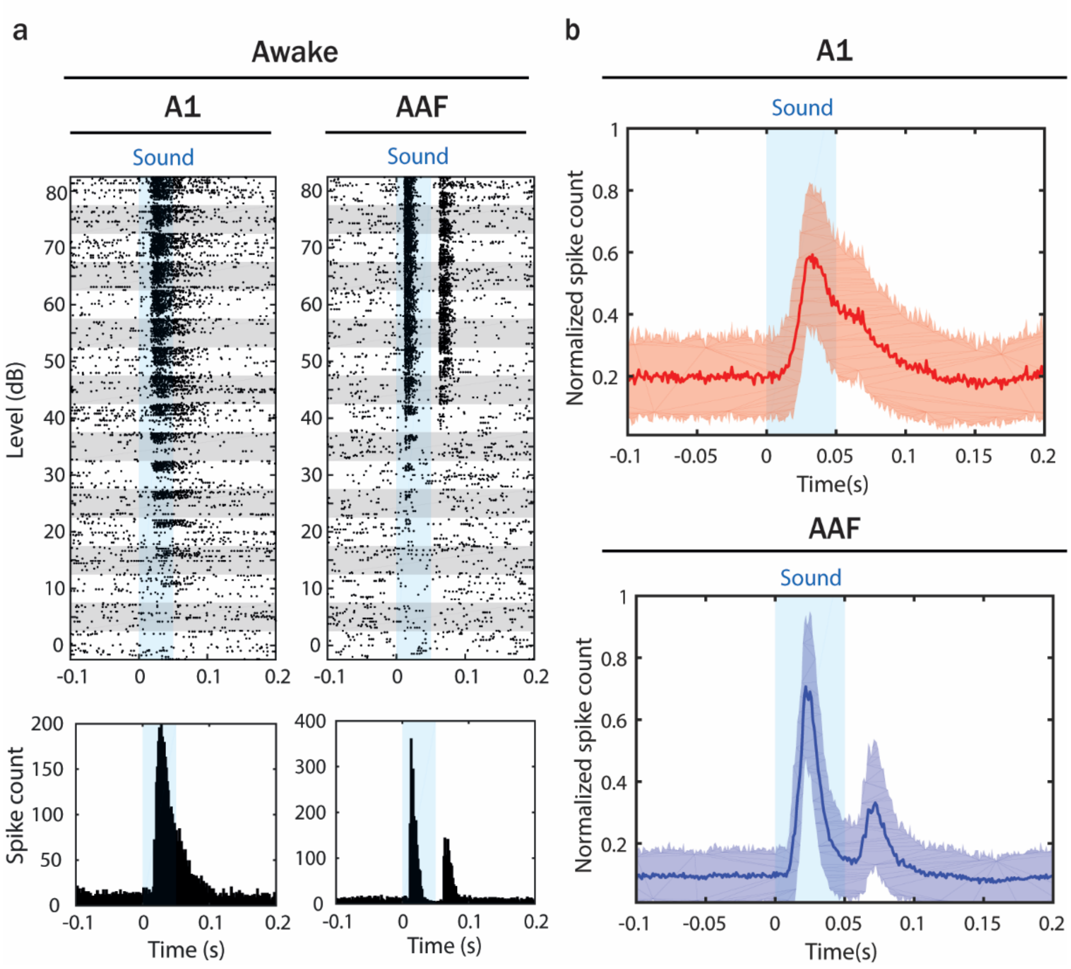

Supplementary Figure 3 Prominent offset responses in AAF are not induced by anesthesia. (**a**) Raster plot and peristimulus time histogram (PSTH) of an example A1 (left) and AAF (right) neuron's response to PTs (tone duration: 50 ms, frequency varying between 4 - 48.5 kHz in 0.1 octave increments, sound level varying between 0-80 dB SPL in 5 dB increments, inter-stimulus-intervals randomized between 500 – 2000 ms) recorded in awake animals. The blue bar represents the tone. (**b**) Averaged PSTH of A1 (top, n=104) and AAF (bottom, n=173) neuron's response to PTs (same sound stimuli as in a) collapsed across frequencies and sound levels.

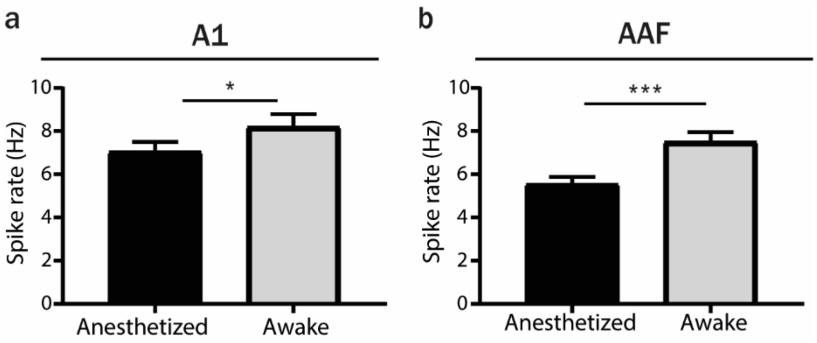

Supplementary Figure 4 Offset responses increase in awake states as compared to anaesthesia. (**a, b**) Comparison of average offset spike rate in A1 (a) and AAF (b) neurons in anesthetized and awake conditions evoked by 50 ms pure tones with frequency varying between 4 - 48.5 kHz in 0.1 octave increments, sound level varying between 0 80 dB SPL in 5 dB increments, inter-stimulus-intervals randomized between 500 – 2000 ms. Data represent mean ± SEM. A1: *p=0.038, anesthetized n=191, awake n=104, AAF ***p=0.0008, anesthetized n=190, awake n=173 , Mann-Whitney test. Higher spike rates at the offset in A1 result from sustained onset responses, which overlap with the offset response analysis window.

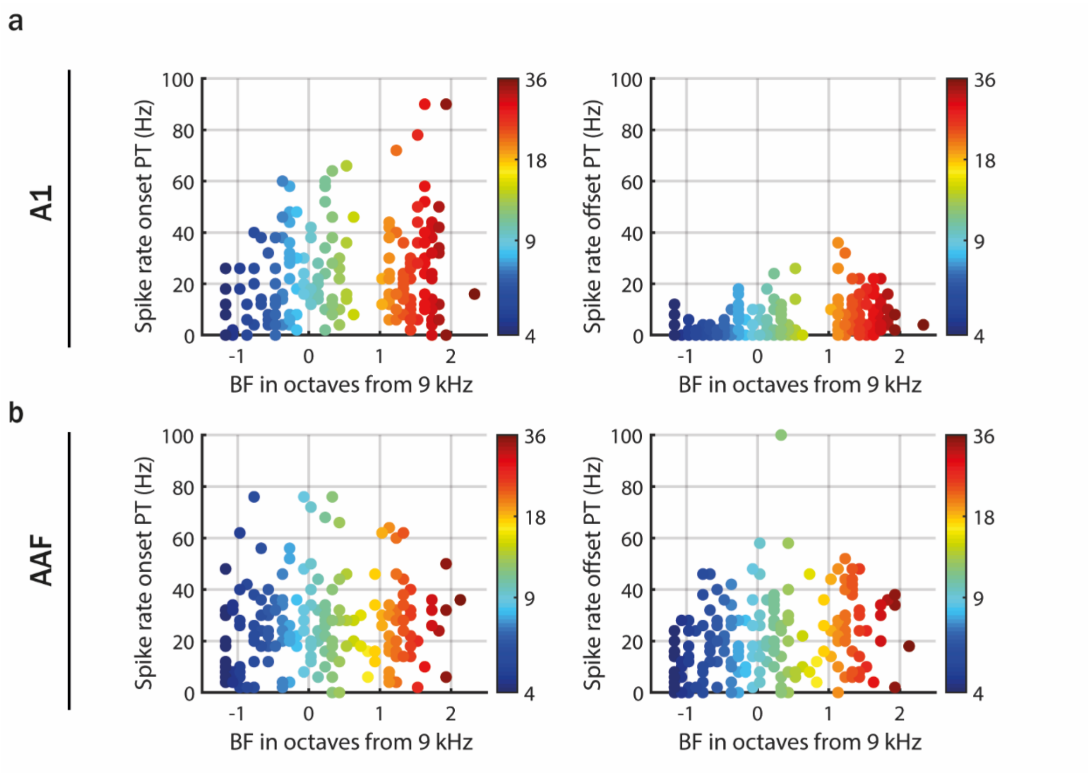

Supplementary Figure 5 Onset and offset responses evoked by 9 kHz PT in A1 and AAF neurons tuned to different frequencies. (**a, b**) Onset (left) and offset (right) responses in (**a**) A1 (n=191) and (**b**) AAF (n=190) as a function of distance of BF from 9 kHz (stimuli used in experiments). Responses are color-coded to neuron's onset BF.

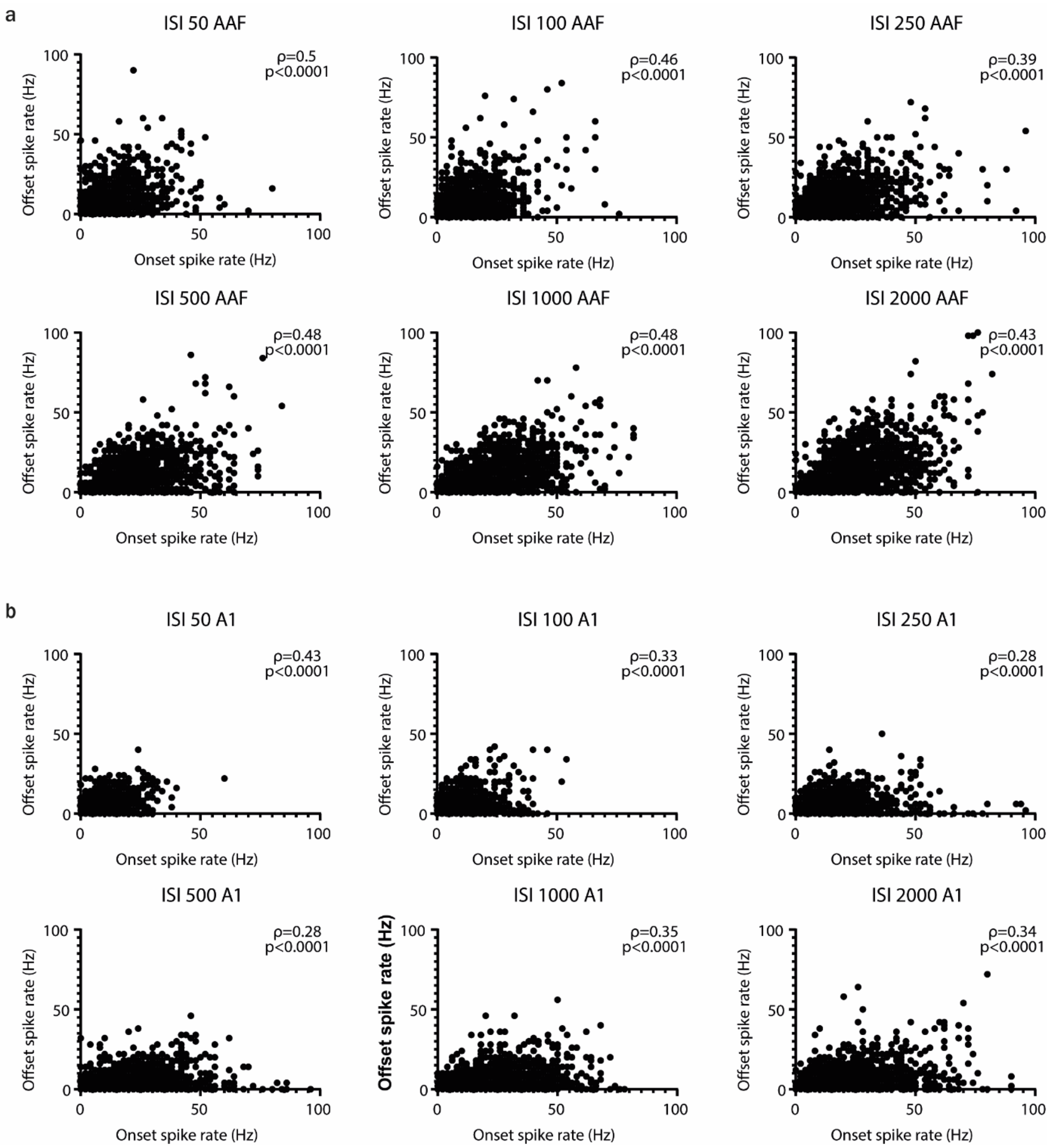

Supplementary Figure 6 Offsets in AAF do not suppress a following onset response. (**a, b**) Correlation between offset and onset responses at each ISI in (**a**) AAF and (**b**) A1 neurons. AAF: n=190, A1: n=191, Spearman correlation.

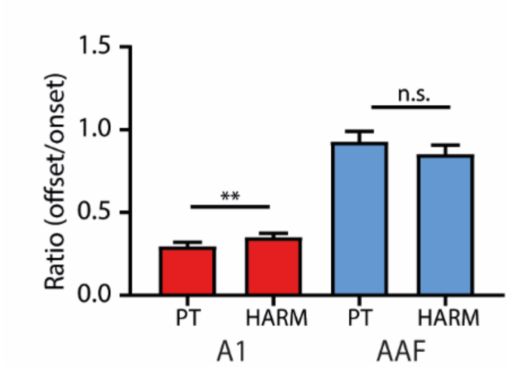

Supplementary Figure 7 Offset responses in A1 are bigger for harmonic than for pure tones. Firing rates of offset relative to onset responses evoked by PTs and HTs in A1 and AAF neurons. Data represent mean ± SEM. A1: **p=0.0035, n=184, AAF: p=0.14, n=187, Wilcoxon Test.

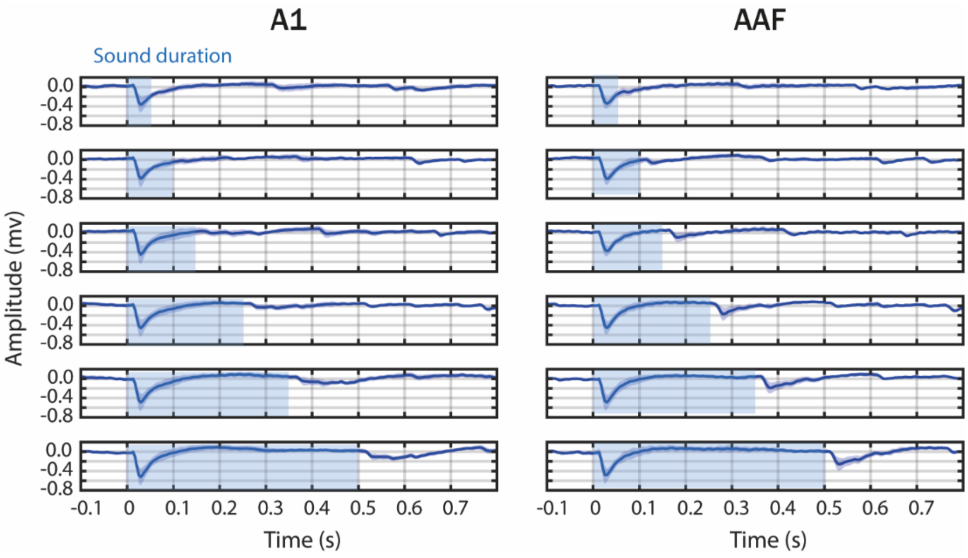

Supplementary Figure 8 Averaged LFP signal from L4 A1 (6 animals) and AAF (7 animals) neurons in response to HTs (9+18+27+36 kHz) played at 60 dB SPL with sound duration varying between 50 and 500 ms and ISI between 50 and 2000 ms. The blue shaded bars represent the tone.

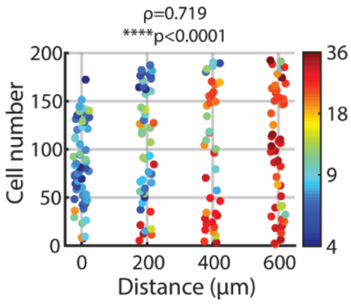

Supplementary Figure 9 Confirmation of tonotopy in A1. Comparison of onset BF of A1 neurons, displayed as a relative distance between electrode shafts. Responses are color-coded to onset BF (correlation between BF and relative distance between electrode's shaft: ρ=0.719, ****p<0.0001, n=191, Spearman correlation).
